# Modulation of protease-activated receptor expression by *Porphyromonas gingivalis* in human gingival epithelial cells

**DOI:** 10.1186/s12903-015-0105-8

**Published:** 2015-10-17

**Authors:** Diya Zhang, Shenglai Li, Lingjing Hu, Lieping Sheng, Lili Chen

**Affiliations:** Dental Department, Sir Run Run Shaw Hospital, School of Medicine, Zhejiang University, Hangzhou, 310016 China; Department of Oral and Maxillofacial Surgery, Stomatology Hospital, School of Medicine, Zhejiang University, Hangzhou, 310006 China; Department of Oral Medicine, The Second Affiliated Hospital, School of Medicine, Zhejiang University, Hangzhou, 310009 China

**Keywords:** Protease-activated receptor, Human gingival epithelial cells, Porphyromonas gingivalis, *P*roteases

## Abstract

**Background:**

Protease-activated receptors (PARs) are G-protein-coupled receptors with an active role in mediating inflammation, pain and other functions. The oral pathogen *Porphyromonas gingivalis* (*P. gingivalis*) secretes proteases that activate PARs. The aim of this study was to elucidate the role of PARs in the pathogenesis of chronic periodontitis by expression analysis of PARs in human gingival epithelial cells (GECs) before and after *P. gingivalis* supernatants treatment.

**Methods:**

GECs were isolated from healthy human gingival tissue samples. The expression of PARs in GECs was determined by reverse transcription-polymerase chain reaction (RT-PCR) and flow cytometry. The effect of *P. gingivalis* proteases was investigated by quantitative real-time reverse transcription polymerase chain reaction (QRT-PCR) and flow cytometry.

**Results:**

PAR-1, PAR-2, and PAR-3 were expressed in GECs. PAR-4 was not found by both RT-PCR and flow cytometry. Analysis of gene expression using QRT-PCR showed an up-regulation of PAR-2 mRNA in comparison to the untreated control cells (*P* < 0.05). In contrast, the mRNA expressions of PAR-1 and PAR-3 were significantly down-regulated (*P* > 0.05) in response to *P. gingivalis* supernatant compared to that in unstimulated control cells. This effect was abrogated by the protease inhibitor TLCK (*P* < 0.05). The results of flow cytometry indicated PARs protein levels consistent with mRNA levels in the results of QRT-PCR.

**Conclusions:**

Our study shows that PAR-1, PAR-2 and PAR-3 are expressed in GECs. *P. gingivalis* proteases play a role in the regulation of innate immune responses in GECs. GECs use PARs to recognize *P. gingivalis* and mediate cell responses involved in innate immunity.

## Background

Protease-activated receptors (PARs) are a subfamily of G protein-coupled receptors (GPCRs) with four members, PAR-1, PAR-2, PAR-3 and PAR-4, which play critical roles in hemostasis, thrombosis, embryonic development, wound healing, inflammation and cancer progression [1]. The activation of PARs occurs by a unique mechanism that involved specific proteolytic cleavage of the N-terminal extracellular sequence by a protease. This cleavage unmasks a new N-terminal sequence, which acts as a tethered ligand that binds to the receptor to initiate multiple signaling cascades [2–4]. Although they share the same mechanism of action, it has been shown that different PARs are characterized by different distributions and biological actions and can be activated by different proteases [5]. Thrombin is a major activator of PAR-1, PAR-2 and PAR-3. Other important activators of PAR-1 include activated protein C (APC) and matrix metalloproteinase-1 (MMP-1); whereas trypsin and human mast cell tryptase activate PAR-2 and trypsin and cathepsin G activate PAR-4. In terms of eliciting downstream signaling responses, PAR-1, PAR-2 and PAR-4 can signal autonomously, while PAR-3 is mainly considered to be a co-receptor for PAR-1 and PAR-4 [6–9]. As PARs are expressed in a wide variety of cell types, it has recently been suggested that they play important roles in physiological processes, such as growth, development, inflammation, tissue repair and pain.

The gingival epithelium is becoming known as a regulator of the oral innate immune response to a variety of insults, such as bacteria and chemicals. Human gingival epithelial cells (GECs), which are key factors of innate immunity, not only play an important role in maintaining the physical barrier between the host and the environment but also actively participate in tissue innate immunity [10, 11] by specifically expressing certain receptors that are involved in the host immune response. It is widely accepted that cells utilize pattern recognition receptors to identify bacteria in their environment [12, 13]; however, in addition, some pathogens, such as *Porphyromonas gingivalis* (*P. gingivalis*), secrete proteases that are recognized by cells via the family of PARs [14]. PARs are known to be expressed in the epithelium of gingiva and have been implicated in the pathogenesis of periodontitis [15–18].

Periodontitis is a chronic infectious disease that starts as inflammation of the periodontal tissues and finally causes resorption of alveolar bone and subsequent loss of teeth. *P. gingivalis* is a major causative agent of chronic periodontitis. This bacterium produces and releases a large amount of proteolytic enzymes. Trypsin-like proteinases, called gingipains, produced by *P. gingivalis* have been shown to act as important pathogenic agents [19, 20]. Recently, it has been shown that gingipains are recognized by cells via the family of PARs, which are involved in inflammatory processes in several tissues. However, the precise roles of PARs in gingival tissues and the importance of specific PARs in the pathogenesis of periodontitis remain to be elucidated. In the present study, reverse transcription-polymerase chain reaction (RT-PCR) was used to investigate the mRNA levels of PARs and flow cytometry was used to investigate the protein levels of PARs in GECs. Furthermore, quantitative real-time RT-PCR (QRT-PCR) was used to investigate the mRNA levels of PARs in response to cell-free supernatant from *P. gingivalis* in order to corroborate the roles of the secreted proteases.

## Methods

### Gingival epithelial cell culture

Primary human GECs were isolated from healthy human gingival tissue samples from patients undergoing third molar extraction at the Dental Department, Sir Run Run Shaw Hospital, School of Medicine, Zhejiang University, China. Written informed consent was obtained from all individuals participating in this study. The study were evaluated and approved by the Ethics Committee of the Affiliated Sir Run Run Show Hospital of Zhejiang University School of Medicine (20131120). Fresh gum tissue was placed into D-Hanks containing 300 U/ml penicillin G and 300 μg/ml streptomycin and incubated at 4 °C. Within 1 h, tissue was prepared to obtain epithelial cells. Briefly, the tissue was cut into small pieces (1 mm × 1 mm), treated with a solution of 25 % dispase II (Sigma–Aldrich, St Louis, MO, USA) and incubated for 18 h at 4 °C. After incubation, the epidermal layer of human keratinocytes was lifted from the dermis and placed into a 15 ml sterile centrifuge tube containing 2 ml trypsin–EDTA. The tissue was incubated at 37 °C for approximately 10 min. Subsequently, isolated GECs were seeded into T-75 flasks (BD Biosciences) at a cell density of approximately 3 × 10^6^ cells per flask in 10–15 ml serum-free keratinocyte medium (keratinocyte-SFM) to which supplements were added according to the manufacturer’s instructions (Gibco BRL, Life Technologies, Rockville, MD, USA). Fluids in the flasks were exchanged for fresh complete medium and gassed with 5 % CO_2_ every 2–3 days. Cells were passaged when 75–80 % confluence was reached.

### Reverse transcription-PCR (RT-PCR) analysis for the determination of PARs expression

To examine the expression of PARs mRNA, total RNA was isolated from GECs (grown to 70 % confluence) using TRIzol® reagent (Gibco BRL, Life Technologies, Rockville, MD, USA) according to the manufacturer’s suggested protocol. The synthesis of the first strand cDNA and RT-PCR were performed using a PromeScript® RT-PCR Kit (Takara Biotechnology Co., Ltd, Dalian, China). The primers for PAR-1, PAR-2, PAR-3, PAR-4 and β-actin were synthesized by Sangon Biotech Co., Ltd (Shanghai, China; Table 1). For amplification of PAR-1, PAR-2 and β-actin products, PCR was performed for 30 cycles. The first cycle included a denaturation step of 5 min at 94 °C. Cycles 2–30 had a denaturation step of 30 s at 94 °C, 30 s of annealing at 60 °C and 45 s of elongation at 72 °C. The last cycle included an elongation step of 10 min at 72 °C. For PAR-3 and PAR-4 amplification, PCR was performed for 30 cycles. The first cycle included a denaturation step of 5 min at 94 °C. Cycles 2–30 had a denaturation step of 30 s at 94 °C, 30 s of annealing at 65 °C and 45 s of elongation at 72 °C. The last cycle included an elongation step of 10 min at 72 °C. DNA products and molecular weight marker DL1,000™ DNA Marker (Takara Biotechnology Co., Ltd, Dalian, China) were separated in 1.5 % agarose gel, after which the gels were stained with GelRed™ and visualized under UV light.

**Table 1:** Oligonucleotide sequences used for RT-PCR

Primers	Oligonucleotide sequence		Length (bp)
	Sense (5’ to 3’)	Antisense (5’ to 3’)	
PAR-1	CCCGCAGGCCAGAATCAAA	AAAGGGGAGCACAGACACAAACAG	395
PAR-2	CTACTCAGATGACCCCAGAAACT	CCCAAAGTGCTAGGATTACAGG	399
PAR-3	GGCTGGACAGGAGCCACGAT	AGCGGTTGATGCTGATGCAGG	403
PAR-4	GGATCGCCTACCACCTGCGTG	CCCGTAGCACAGCAGCATGG	401
β-actin	AGGGGCCGGACTCGTCATACT	GGCGGCAACACCATGTACCCT	202

### Bacteria culture and supernatants collection

*P. gingivalis* ATCC 33277 was purchased from the American Type Culture Collection (Manassas, VA, USA) and anaerobically cultured (80 % N_2_, 10 % H_2_ and 10 % CO_2_) in a brain–heart infusion (BHI; Oxoid) agar plate containing 5 % defibrinated sheep blood enriched with 5 g/l yeast extract, 5 mg/l hemin and 10 mg/l menadione (Sigma–Aldrich, Dorset, UK) at 37 °C for up to 5 weeks. Liquid cultures were prepared by inoculation of bacterial colonies (3–4 days old) from blood agar plates into 10 ml BHI broth supplemented with 5 g/l yeast extract, 5 mg/l hemin and 10 mg/l menadione and incubated for 24 h. Ten percent inoculum was transferred to 90 ml of the same medium and incubated for 6 days. After this culture period, bacteria were harvested by centrifugation at 10,000 *g* for 15 min at 4 °C and supernatants were collected, filter-sterilized over a 0.2 mm filter and stored at −80 °C until use. Before treatment, aliquots of the supernatant were used for pre-incubation (10 min) with 1 mmol/l of the serine and cysteine protease inhibitor tosyl-L-lysine chloromethylketone (TLCK; Sigma–Aldrich, St Louis, MO, USA), which inhibits gingipains [15, 21].

### Characterization of bacterial culture supernatants and treatment

*P. gingivalis* supernatants were diluted in cell culture medium and their concentration expressed as the total bacterial protein (mg/ml) present in the cell cultures. The protein concentration was determined with a BCA Protein Assay Kit (Pierce, Rockford, IL, USA). Absorbance was measured at 562 nm on a SpectraMax® Plus plate reader. Protease activity was measured with a Protease Assay™ Kit (G-Biosciences, St Louis, MO, USA) [22]. The absorbance of the dye-labeled peptide was measured at 570 nm for determination of the protease activity. Chemically stabilized trypsin (MSG-Trypsin™) was supplied with the kit as a general protease standard. GECs were grown to 80 % confluence and stimulated with either 50 μg/ml culture supernatant protein from *P. gingivalis* supernatants or TLCK-preincubated supernatants, for 6 h [22]. Unstimulated GEC medium served as a control for the stimulation experiments. Each stimulation experiment was performed in triplicate and cells from two to five different donors were tested.

### Quantitative real-time RT-PCR (QRT-PCR)

After stimulation, total RNA was extracted using an RNeasy Kit (Qiagen, Valencia, CA, USA) and reverse-transcribed using a SuperScript® RT-PCR Kit (Takara, Tokyo, Japan). Quantitative real-time RT-PCR was performed using the Applied Biosystems 7500 PCR machine and SYBR Premix Kit (Invitrogen, Carlsbad, CA, USA) according to the manufacturer’s instructions. PCRs were carried out in 96-well plates in a total volume of 20 μl, including 1 μl cDNA and 0.8 μl primers (10 μM; Table 2). Sample expression was normalized against that of the housekeeping gene β-actin, which was included in each QPCR run. PCR controls were performed using water instead of cDNA. All reactions were carried out in duplicate. At each time point, the expressions of the selected mRNAs in cells incubated with *P. gingivalis* supernatants or TLCK-preincubated supernatants were calculated relative to the housekeeping gene β-actin (Δ*C*_t_) for each sample and then expressed relative to untreated cells at the same time point using the 2^-ΔΔ*C*t^ method [23].

**Table 2:** Oligonucleotide sequences used for QRT-PCR

Gene product	Oligonucleotide sequence	
	Sense (5’ to 3’)	Antisense (5’ to 3’)
PAR-1	GTGATTGGCAGTTTGGGTCT	GCCAGACAAGTGAAGGAAGC
PAR-2	CCTGGCCATGTACCTGATCT	GACACTTCGGCAAAGGAGAG
PAR-3	GGTGTGGGCAACAGTTTTCT	GGACTCGCAAGTGTTGTGAA
β-actin	AGGGGCCGGACTCGTCATACT	GGCGGCAACACCATGTACCCT

### Flow cytometry

Cells were washed in phosphate-buffered saline (PBS), incubated for 30 min at 4 °C with PBS containing 20 % heat-inactivated normal human serum, washed again and then incubated for 30 min at 4 °C with 15 nM of specific monoclonal antibodies (mAb) to human PAR-1–4 (PE-conjugated; Santa Cruz Biotechnology, Inc., Santa Cruz, CA, USA). Flow cytometry analyses were performed on a FACScan (BD Biosciences, San Jose, CA, USA). Control cells incubated with PE-conjugated nonspecific antibodies obtained from the same manufacturers were used to set the threshold for the fluorescence parameter, such that the fraction of cells with positive fluorescence was <2.5 % of the total cells. The percentage of PAR-1–4 positive cells was determined from the fraction of cells in the sample incubated with specific antibodies that exceeded the threshold for the fluorescence signal intensity obtained with the control sample.

### Statistical analyses

All data are shown as the mean ± the standard deviations (SD). QRT-PCR data are expressed as *C*_t_ (cycle threshold), Δ*C*_t_ (*C*_t_ PAR mRNA – *C*_t_ β-actin mRNA) and relative quantification (RQ; expressed as fold change). The fold changes of PARs mRNA expression were calculated using the 2^-ΔΔ*C*t^ method [23]. Student’s *t*-test was used for comparison between two groups. SPSS version 19.0 (SPSS Inc., Chicago, IL, USA) was used for statistical analysis and *P* ≤ 0.05 was considered statistically significant.

## Results

### RT-PCR analysis of PARs in GECs

**Fig. 1:**
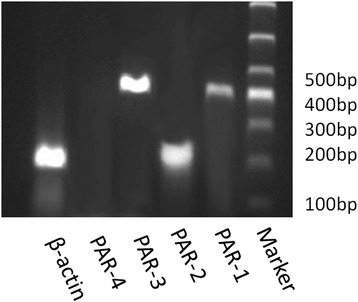
RT-PCR analysis of PARs mRNA in GECs. RT-PCR was performed using the primers specific to each type of PAR, as described in Table 1. The PCR products were subjected to electrophoresis through a 1.5 % agarose gel. Lines 1–6 represent β-actin, PAR-4, PAR-3, PAR-2, PAR-1 and Marker separately. RT-PCR analysis revealed the presence of PAR-1, PAR-2 and PAR-3 mRNA in GECs. No PCR product was found for PAR-4

RT-PCR analysis of RNA extracted from GECs revealed the presence of PAR-1, PAR-2 and PAR-3 mRNA (Fig. 1). No PCR product was found for PAR-4 (Fig. 1). The result shows that PAR-1, PAR-2 and PAR-3 are expressed in GECs, but PAR-4 is not.

### *P. gingivalis* supernatant alters PARs gene expression

**Fig. 2:**
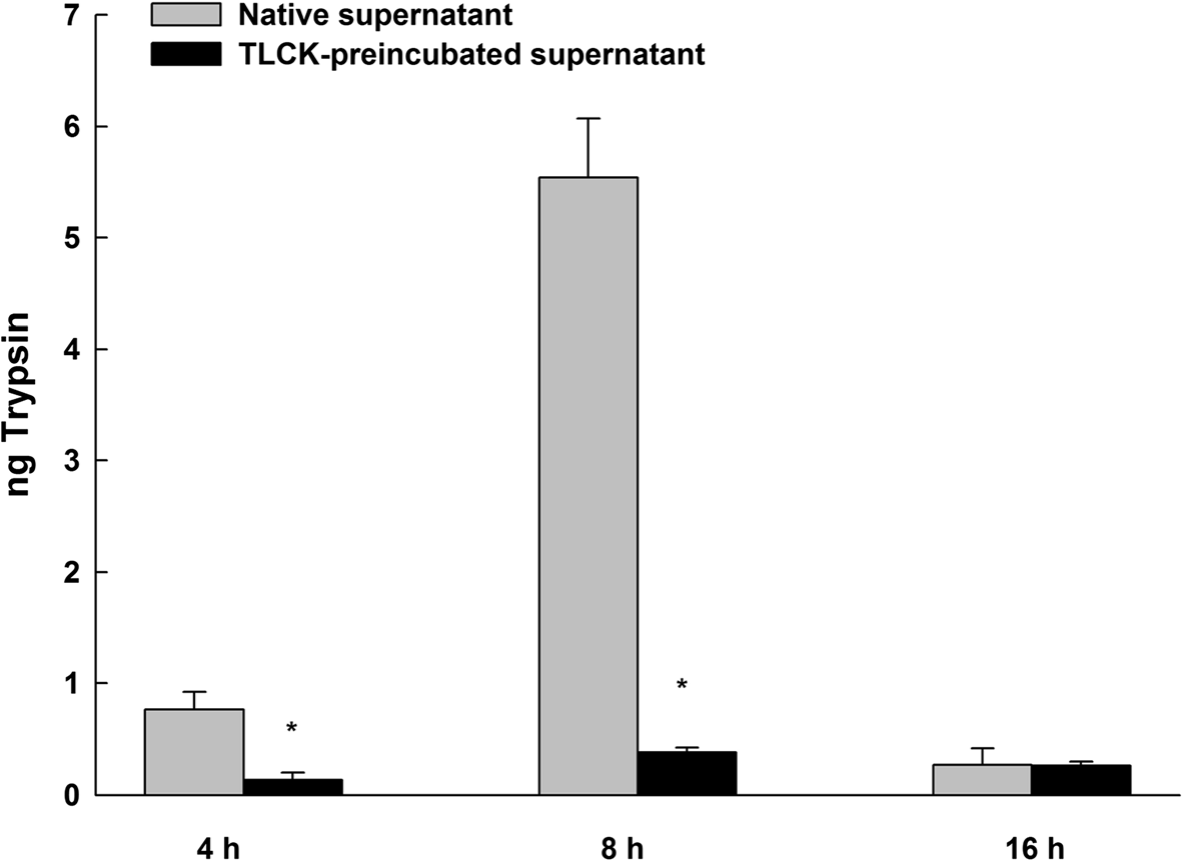
The protease activities of *P. gingivalis* supernatant or TLCK-preincubated supernatant. Protease activity was measured with a Protease Assay™ Kit and was monitored for 4, 8 and 16 h. The absorbance of the dye-labeled peptide was measured at 570 nm for determination of the protease activity. Native supernatant showed significantly higher proteolytic activity than TLCK-preincubated supernatant after 4 and 8 h. Triplicate measurements were performed. Data are shown as the mean with the standard deviation (mean ± SD). *, *P* < 0.05, compared with native supernatant values

**Fig. 3:**
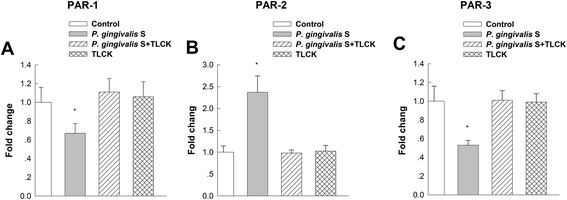
PAR-1 (**a**), PAR-2 (**b**) and PAR-3 (**c**) gene expression in response to *P. gingivalis* supernatant. PARs gene expression was evaluated by quantitative real-time RT-PCR (QRT-PCR) in response to cell-free supernatant from *P. gingivalis* and TLCK-preincubated supernatant in GECs. After *P. gingivalis* supernatant treatment, the expression of PAR-2 was upregulated compared to untreated control cells. Conversely, PAR-1 and PAR-3 expression was significantly downregulated. Data are shown as the mean with the standard deviation (mean ± SD). *, *P* < 0.05, compared with control values. Control: without supernatant stimulation. *P. gingivalis* S: *P. gingivalis* supernatant

The proteolytic activity of the supernatant from *P. gingivalis* was demonstrated after 4, 8 and 16 h. Preincubation of the supernatant with the protease inhibitor TLCK showed significantly reduced proteolytic activity compared to that in native supernatant after 4 and 8 h (*P* < 0.05; Fig. 2). To evaluate the effect of the *P. gingivalis* supernatant on the expression of PAR-1, PAR-2 and PAR-3 mRNAs, GECs were grown to 80 % confluence and stimulated with the supernatant for 6 h. Analysis of gene expression using QRT-PCR showed upregulation of PAR-2 mRNA in comparison to untreated control cells (*P* < 0.05; Fig. 3b). In contrast, the mRNA expressions of PAR-1 and PAR-3 were significantly downregulated (*P* < 0.05) in response to *P. gingivalis* supernatant compared to that of unstimulated control cells (Fig. 3a, c). Preincubation of the *P. gingivalis* supernatant with the protease inhibitor TLCK abolished the effect and restored the PARs mRNA expression levels to that of untreated cells. Controls establish using blank bacterial medium and TLCK showed no effect on the mRNA expression of PAR-1, PAR-2 and PAR-3 (Fig. 3). The results were consistent with previous studies [22].

### PARs protein expression assessed by flow cytometry

**Fig. 4:**
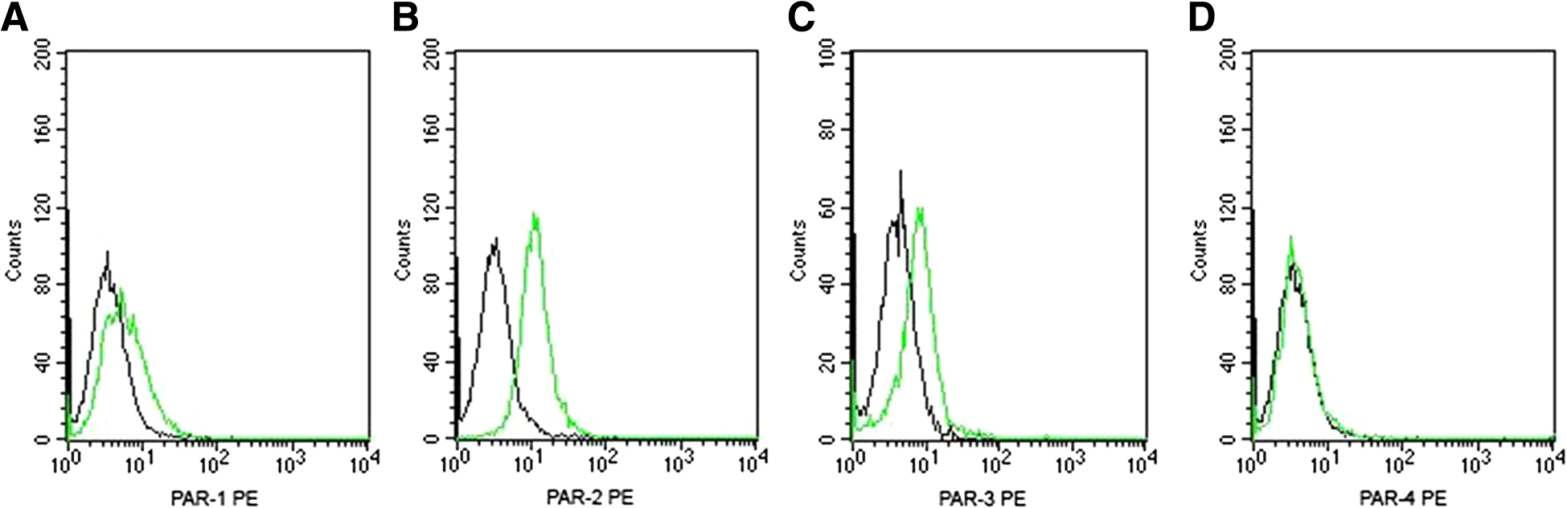
Protein level of PARs in GECs by flow cytometry. **a**, **b**, **c** and **d** show representative results from the flow cytometric analyses of PAR-1, PAR-2, PAR-3 and PAR-4, respectively. The result showed GECs expressed PAR-1, PAR-2 and PAR-3, but not PAR-4. Black line: isotype control. Green line: anti-PARs

**Fig. 5:**
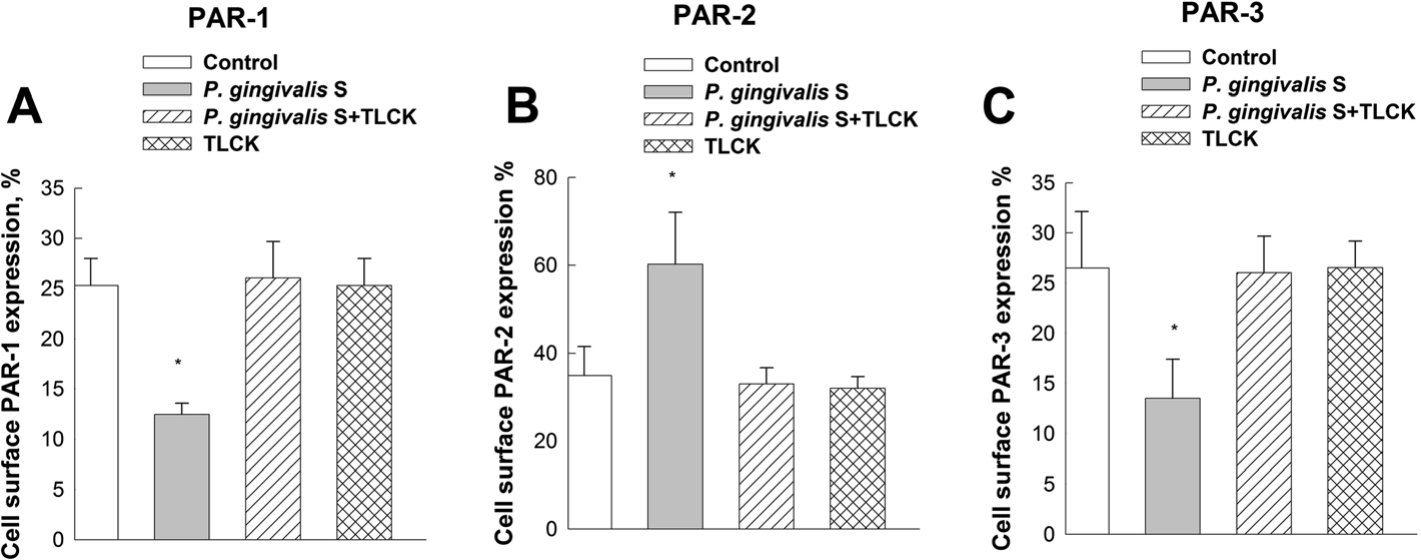
PAR-1 (**a**), PAR-2 (**b**) and PAR-3 (**c**) protein expression in response to *P. gingivalis* supernatant. PARs expression was evaluated by flow cytometry in response to cell-free supernatant from *P. gingivalis* and TLCK-preincubated supernatant in GECs. Flow cytometry analyses were performed on a FACScan. After *P. gingivalis* supernatant treatment for 6 h, the expression of PAR-2 was upregulated compared to untreated control cells. Conversely, PAR-1 and PAR-3 expression was significantly downregulated after treatment. Data are shown as the mean with the standard deviation (mean ± SD). *, *P* < 0.05, compared with control values. Control: without supernatant stimulation. *P. gingivalis* S: *P. gingivalis* supernatant

Flow cytometry showed protein expression of PAR-1, PAR-2 and PAR-3 by GECs, but no PAR-4 expression (Fig. 4). After *P. gingivalis* supernatant treatment for 6 h, the protein expression of PAR-2 was upregulated compared to untreated control cells (*P* < 0.05; Fig. 5). Conversely, the protein levels of PAR-1 and PAR-3 were significantly downregulated after treatment (*P* < 0.05). The results of flow cytometry indicated PARs protein levels consistent with mRNA levels in the results of QRT-PCR.

## Discussion

Periodontitis is an infection of periodontal tissues which are the supportive structure for the teeth. In the complex structure of periodontal tissues, gingival epithelium is directly exposed to periodontal bacteria and their products, by receiving and transmitting signals, plays an important role in the overall dialogue that occurs between pathogens and the host. PARs are expressed and function on GECs as a part of the immuno-surveillance system. These receptors are clearly important for GECs responses to the environment that may include pathogenic or physiological products.

Four PAR family members (PAR-1, −2, −3, and −4) have been identified so far. In this study we showed that PAR-1, PAR-2 and PAR-3 are expressed in GECs, but PAR-4 is not expressed in GECs. Many researchers have already reported the expression of PARs in a wide variety of cell types, while there was controversy about the expression of PARs [2, 3, 13, 17]. There are also some studies about the expression of PARs in GECs, but most studies were concerned on the expression and the roles of PAR-2. Overexpression of PAR-2 has been positively associated with inflammatory clinical parameters and with the levels of interleukin-6 (IL-6), IL-8, tumor necrosis factor alpha, matrix metalloprotease-2 (MMP-2), MMP-8, hepatocyte growth factor and vascular endothelial growth factor [5, 24, 25]. Only one research found PAR-1, PAR-2 and PAR-3 expression in KB cells by RT-PCR and PAR-1, PAR-2 and PAR-3 expression in GECs by immunohistochemical studies [17]. In our study, RT-PCR and flow cytometry were used to analyse the expression of PARs. The results were corroborated the PAR-1, PAR-2 and PAR-3 expression in GECs.

In gingival epithelium, arginine-specific cysteine proteases (Arg-gingipain, Rgp) from *P. gingivalis*, a major pathogen associated with chronic periodontitis, activate PARs and induce expression of inflammatory cytokines [22, 26, 27]. The PAR family is structurally unrelated to the pattern recognition receptor family (PRR) but, like PRRs, they signal potential danger in the environment by activating innate immune markers and inflammatory responses [28]. However, little is known about their function when they are activated by their agonist enzymes, thrombin and trypsin or trypsin-like enzymes, in gingival epithelium. PARs are expressed by a wide variety of cell types and are suggested to play important roles in physiological processes, such as growth, development, inflammation, tissue repair and pain [29]. Our study demonstrated that the expression of PAR-2 was upregulated with *P. gingivalis* supernatant treatment compared to untreated control cells. Conversely, PAR-1 and PAR-3 expression was significantly downregulated, after *P. gingivalis* supernatant treatment. In fact, PAR-2 activation has been associated with several chronic inflammatory conditions [3, 30–32]. Furthermore, in vitro and in vivo studies have clearly suggested that PAR-2 also plays a role in periodontal inflammation [15, 16, 33, 34]. PAR-1 activation, via thrombin or man-made activating peptides, has been implicated as a potential regulator of both pain and inflammation [35–37]. A recent study also showed that PAR-1 was expressed in gingival tissues [18] and PAR-1 activation by thrombin may play a role in the repair and homeostasis of periodontal tissues [38]. The role of PAR-3 is of interest because the function of this apparently non-signaling receptor remains obscure [9, 39, 40]. The ability of PAR-3 to generate an intracellular signal also remains in doubt because it lacks the cytoplasmic tail domain shown in other PARs, which is required to couple with G-proteins [9]. A recent study has demonstrated that PAR-3 is a critical determinant of PAR-1 function and PAR-3 may mitigate the effects of PAR-1 in activating endothelial responses, such as vascular inflammation [41]. PAR-3 regulates PAR1 signaling by receptor dimerization. There has been controversy over the role of PARs. In some tissues they play a proinflammatory role [42], while in other tissues they seem to have an anti-inflammatory or protective effect [43]. This protective function may be facilitated by the upregulation of PAR-2 and the downregulation of PAR-1 and PAR-3 gene expression in response to *P. gingivalis* proteases. However, further studies are required to fully elucidate the roles of PAR-1, PAR-2, and PAR-3 in the pathogenesis of periodontitis.

## Conclusions

The study described here confirms the roles of PAR-1, PAR-2 and PAR-3 in gingival epithelial responses that may be related to periodontal inflammation. Consequently, the detailed mechanisms underlying the effects mediated by the major PARs in periodontitis, including their correlation with cytokines, require further research. This information will provide a better understanding of the development of periodontal diseases and inform the strategy for the identification of therapeutic approaches for these conditions.
